# Chloroquine and hydroxychloroquine for COVID-19: time to close the chapter

**DOI:** 10.1136/postgradmedj-2020-138585

**Published:** 2020-08-11

**Authors:** Anunay Gupta, Amit Malviya

**Affiliations:** Cardiology, Vardhman Mahavir Medical College and Safdarjung Hospital, New Delhi, India; Cardiology, North Eastern Indira Gandhi Regional Institute of Health and Medical Sciences, Shillong, India

COVID-19 pandemic has brought about a surge in repurposing of drugs, either for the treatment of severe acute respiratory syndrome coronavirus 2 (SARS-CoV-2) infection or for the pre-exposure and post-exposure prophylaxis. Many drugs are being tried, but 4-aminoquinolines (chloroquine and hydroxychloroquine) have attracted significant attraction and generated the maximum controversy. There is no domain left, be it political, social or scientific, where usage of 4-aminoquinolines for COVID-19 was undisputed. So far, the benevolent and propitious story of hydroxychloroquine is tainted by political controversies, death threats to researchers and scientific lapses.^1–4^ A significant part of this story appears to be fuelled with the fear generated by the current pandemic, lay media perusal, amplification by social media and political pressure rather than true scientific approach.^4 5^ At the ground level, self-medication and stockpiling are resulting in unavailability for those who really need it,^6 7^and millions of people are exposed to its rare but potentially serious adverse effects including cardiac arrhythmias. As healthcare providers and as a scientific community at large, our fundamentals first guide us to do no harm to the people whose healthcare is in our hands. The dire need to quickly develop, assess and adopt medications during a public heath crisis can go off-centre at times. There has been an enormous discussion over the appropriate usage of this medication and at present, the bone of contention, is whether physicians should prescribe them or not? There indeed, is an urgency of care in ongoing novel coronavirus pandemic and physicians need to decide about appropriateness of this therapy.

But now the time has come to close the chapter of controversies, because enough scientific evidence has accumulated to answer three basic logical questions relating to usage of chloroquine and hydroxychloroquine for treatment and prophylaxis of COVID-19. First, what are the specific mechanisms (in vitro effects) of 4-aminoquinolines on SARS-CoV-2? Second, what does the data say about its efficacy in properly conducted randomised control trials? And finally, what is the safety profile of this drug in specific reference to COVID-19?

4-Aminoquinolines have inhibitory in vitro effects on the replication of SARS-CoV-2, but these mechanisms are a part of its broad antiviral and immunomodulatory properties, and no specific mechanisms are described.^8 9^ In this context, it essential to mention that complex pharmacokinetics of these drugs makes it difficult to extrapolate laboratory dosing and drug concentrations in human subjects.^10^ Moreover, for the patients, clinical outcomes are more important than viral clearance in the laboratories. Experience and evidence from the past indicate that in vitro efficacy of majority of the molecules does not replicate in biological systems.

There are numerous studies including randomised trials on this subject, but they are marked either by poor study design, low sample size, unvalidated end points, substantial confounders or insufficient data^11–14^ and they cannot be relied upon to reach any meaningful conclusion. However, there are studies with proper methodology and reliable data. From these studies, it can be safely concluded that hydroxychloroquine is not effective as post-exposure prophylaxis, it does not lead to reduction in mortality or need of mechanical ventilation for sick patients and is associated with more adverse events.^15–19^ Recently, large international trials such as RECOVERY(Randomised Evaluation of Covid-19 Therapy ), SOLIDARITY(International trial by World Health Organisation) and DISCOVERY(Trial of Treatments for COVID-19 in Hospitalized Adults) have halted the hydroxychloroquine arm, citing internal and external data showing no benefits with such therapy.^20–22^

As far as safety is concerned, many are advocating this drug as very safe based on safety data from patients with rheumatic diseases, but application of such data for COVID-19 is biologically not plausible. The disease process and its metabolic consequences promote a proarrhythmic milieu, and added side effects of medication can be devastating.^11 23^ In patients with severe COVID-19 with hepatic and renal dysfunction who are administered other medications, the metabolism and clearance of hydroxychloroquine are altered and the safety of hydroxychloroquine is yet to be proven conclusively.

The United States Food and Drug Administration (FDA) has revoked the emergency use authorisation of chloroquine and hydroxychloroquine to treat hospitalised patients with COVID-19. The FDA determined that chloroquine and hydroxychloroquine are unlikely to be effective in treating COVID-19, and serious cardiac adverse events and other potentially serious side effects outweigh the known and potential benefits of chloroquine and hydroxychloroquine.^24^

It can be concluded now that 4-aminquinolines have some in vitro activity on SARS-CoV-2, but its efficacy on human disease is doubtful. It does not work for treatment of severe illness and does not prevent infection after high to moderate risk exposure. Its role in pre-exposure prophylaxis and treatment of mild-to-moderate disease remains to be investigated. As far as pre-exposure prophylaxis is concerned, other preventive methods exist which have proven efficacy,^25^ and exposing a large population to a potentially toxic drug when its benefits are not proven beyond doubts is not justified. The Indian Council of Medical Research recently published a case–control investigation asserting that four or more doses of hydroxychloroquine results in a significant decline in the odds of catching infection.^26^ Moreover, this study is severely limited by its design and case–control methodology. Recent practice guidelines by the American College of Physicians do not recommend chloroquine or hydroxychloroquine either for prophylaxis or treatment.^27^ Treatment of mild or moderate COVID-19 illness responds well to conservative therapy or approved antiviral agents, and the same logic as metioned earlier applies for not using hydroxychloroquine in this subset of patients as well. This becomes even more relevant because RECOVERY trial data have shown mortality benefit with dexamethasone, which is much cheaper, more easily available in and arrhythmic risk is least as compared to hydroxychloroquine. Finally, pursuing and investing in this direction does not appear to be prudent and it is time to close the chapter of 4-aminoquinolines for usage in COVID-19.
